# Supplementary material for: PufCB-Auth: A lightweight continuous multi-factor authentication scheme integrated PUF with charging behavior features for EV charging
Source: PLoS One. 2026 May 15;21(5):e0344506. doi: 10.1371/journal.pone.0344506 (PMC13178865; doi:10.1371/journal.pone.0344506)
Supplement: S1 File — (DOCX) [file pone.0344506.s002.docx]

**Appendix A**

The appendix is user behavior features extraction method. The following key features are extracted from the historical charging data and represented them as vectors:

- Spatio-temporal features:

Charging time distribution: counting the charging frequency of users in different time periods (e.g., early morning, morning peak, holidays). The time preference vector of users in a certain time period is denoted as:

$t=\left\{ t_{1},t_{2},\ldots,t_{k} \right\},\sum_{i=1}^{k} t_{i}=1$

Geo-location preference: records the user's charging frequency at commonly used charging locations (e.g., residential areas, commercial areas, highway service areas). The geographic location preference vector of the user for a certain time period is noted as:

$L=\left\{ l_{1},l_{2},\ldots,l_{r} \right\},\sum_{i=1}^{r} l_{i}=1$

- Charging demand characteristics:

Charge preference: take the mean, *μ_Q_*standard deviation *σ_Q_* and quartiles (*Q_25_*, *Q_50_*, *Q_75_*) of the user's extraction of a single charge to construct a demand vector:

$Q=\left[ \mu_{Q},\sigma_{Q},Q_{25},Q_{50},Q_{75} \right]^{T}$

Charging Frequency: counts the number of times a user charges in a given period *T*, which is used to identify regular or sudden charging behavior of the user. Marked as:

$f=\frac{N_{charge}}{T}$

- Interaction dynamics characterization

Session Interruption Patterns: record the frequency of user-initiated termination of charging or abnormal disconnection to construct a baseline for abnormal interruption detection. If the user has too many interruptions within a certain period of time, it may indicate unstable or suspicious operation. Let's say the user has experienced *Φ* interruptions or abnormal disconnections in the last *S* charging sessions, and the ratio of interruptions can be recorded as

$\Phi_{ratio}=\frac{\Phi}{S}$

Determine whether the interrupt behavior is abnormal or not based on a set threshold *ε_Φ_*, if:

$\varepsilon_{\Phi}>\Phi_{ratio}$

Then it is regarded as unstable or suspicious charging behavior, which in turn triggers the corresponding anomaly detection or secondary authentication process.

To facilitate the subsequent calculation, the above features can be integrated into a multi-dimensional vector, and the values of each of these dimensions can be obtained by aggregating historical charging data.

$B=\left[ t,L,Q,f,\Phi_{ratio} \right]$

**Appendix B**

**Appendix B.1**

The appendix is experimental system configuration. In this study, a Raspberry Pi 4 Model B supporting GPIO and SPI/I2C interfaces is implemented to simulate the interaction process between the On-Board Unit (OBU) and the Charging Entry Unit (CEU), equipped with the components shown in Table B1:

**Table B1. Hardware Configuration of the Experimental Simulation Platform**

| **Component Purpose** | **Model Version** | **Purpose** |
| --- | --- | --- |
| External ADC module | ADS1115 | Voltage acquisition for analog BMS |
| Encryption chip | ATECC608A | secure key storage |
| Temperature sensor | DS18B20 | Analog battery temperature monitoring |
| Batteries | batteries*6 | Analog battery packs |
| GPS module | NEO-6M | Analog vehicle position |
| Real Time Clock (RTC) Module | DS3231 | Ensures time accuracy |
| RC delay circuit |  | Analog PCB signal delay difference |
| Connecting wires, breadboards |  | placing of each component |

The experiments were performed in a constant temperature environment (25°C ± 1°C) to avoid temperature fluctuations affecting the PUF characteristics. A regulated power supply was used to supply power to eliminate the effect of voltage fluctuation on the sampling accuracy of the ADC. Calibrate the gain error of ADS1115 by a standard voltage source (2.5V). Calibrate the linearity of DS18B20 beforehand to ensure accurate temperature acquisition.

**Appendix B.2**

The appendix is what needs to be done to initialize the experiment, and registration data statistics:

Analog PUF data registration: 2 battery channels randomly selected from 4, 2 temperature sensors randomly selected from 3, and one RC circuit randomly selected from 2 as a random challenge. Thirty-six random challenges can be generated as described above and extended to 150 groups by randomizing the perturbation parameters. The hash of the previous response is used as a seed to iteratively generate 50 sets of unpredictable challenges based on the hash chain. After sending challenges to the simulated PUF, the voltage values and ordering of the selected channels are recorded, the noise variance of the two sensors is calculated, and the RC delay is recorded with mean and variance as the corresponding response.

Behavior features data collection: a one-year behavior model is generated to cover typical user habits. A segmented weighted Poisson process is used to change the data generation parameters at different times of the year to simulate the differences in the charging probability of the users at different times of the year, e.g., the charging frequency is appropriately lowered on weekdays, appropriately raised on weekends, and adjusted according to the situation on holidays. In this way, 5 users are generated, and a number of sub-charging records are generated within a year. The charging coordinates are generated according to different ratios, labeled as home, business, shopping mall, and the corresponding data ratios are set for different charging locations, as well as the corresponding coordinate offset values. Each user's charging record is assigned a charging type according to a certain ratio, such as 80% fast charging data and 20% slow charging data, as well as charging power, starting SOC and other related information. Data generation statistics are shown in Table B2

**Table B2. Parameter Settings for Simulated Charging Behavior**

| **Data type** | **Quantity/parameter** | **Description** |
| --- | --- | --- |
| PUF CRP | 200 sets | 150 static CRPs, 50 dynamic CRPs |
| Charging event logging | 5 users, one year duration | Randomized Charging Behavior Parameters by User Category |
