# Supplementary material for: PufCB-Auth: A lightweight continuous multi-factor authentication scheme integrated PUF with charging behavior features for EV charging
Source: PLoS One. 2026 May 15;21(5):e0344506. doi: 10.1371/journal.pone.0344506 (PMC13178865; doi:10.1371/journal.pone.0344506)
Supplement: Attachment — Submitted filename: Response_to_Reviewers.docx [file pone.0344506.s003.docx]

Dear Editor,

We would like to thank the Academic Editor and the reviewers for their valuable comments and feedback that has helped us make improvements to this paper (No. **PONE-D-25-41682**, titled **"PufCB-Auth: A Lightweight Multi-Factor Continuous Authentication Scheme Integrated PUF with Charging Behavior Features for EV-ChaoJi Charging Piles Interaction Scenarios"**). We have carefully revised the manuscript addressing all of the reviewer comments.

We uploaded (a) our point-by-point response to the comments in this response letter and (b) an updated manuscript with highlighted changes.

In this point-by-point response, the editors' and reviewers' comments are in normal black-colored, and our responses are presented in blue-colored. We have indicated key modifications to the revised manuscript in red color.

We thank all the reviewers for their valuable time and constructive comments. We have considered their insightful comments carefully and revised the paper accordingly.

We look forward to your comments,

On behalf of all authors.

Dr. Ji Ma

**Response to Reviewer 1’s Comments**

**Comments:**

**1.0 Response:**

We thank Reviewer #1 for the thorough and insightful review. We address each major and minor comment below.

**Major Comment 1**

The experimental evaluation is insufficient, as the proposed scheme is only compared with its own sub-components (Puf-Auth and CB-Auth). The authors should compare PufCB-Auth with existing state-of-the-art lightweight MFA schemes.

**1.1 Response:**

We sincerely thank the reviewer for this insightful and important comment. We fully agree that comparing the proposed PufCB-Auth scheme only with its internal sub-components (Puf-Auth and CB-Auth) is insufficient to convincingly demonstrate its novelty and practical advantages. Such an evaluation indeed only validates the general benefit of multi-factor authentication over single-factor methods, which is already well established in the literature.

In response to this comment, we have substantially expanded the experimental evaluation and security analysis to include comparisons with representative state-of-the-art lightweight authentication schemes from related EV and IoT scenarios. Our goal is to provide a fair, transparent, and reproducible comparison under unified assumptions, rather than selectively reporting favorable metrics.

Specifically, we have selected two representative baseline schemes that reflect mainstream lightweight authentication paradigms in the literature:

1, PUF-MAP, which represents hardware-centric authentication schemes relying primarily on PUF-based challenge–response mechanisms. This category emphasizes unclonable device identity and low computational overhead but lacks adaptability to user behavior and long-term interaction dynamics.

2, L2AI, which represents behavior-driven authentication schemes that leverage charging or usage behavior for identity verification. This category captures user-level authentication capabilities but does not incorporate hardware-rooted identity and is vulnerable to long-term mimicry.

These two schemes jointly represent the dominant design philosophies in existing lightweight authentication research—hardware-only and behavior-only—thereby serving as appropriate and meaningful baselines for evaluating the proposed multi-factor design.

To ensure comparability, we introduced a unified threat model and security evaluation methodology, explicitly defining the adversarial capabilities (network control, impersonation, partial device compromise, behavior manipulation, and update abuse). Under this unified threat model, we conducted a structured qualitative security comparison, focusing on attack resistance rather than isolated performance metrics.

In addition, we extended the experimental evaluation to include:

A qualitative attack-resistance comparison across multiple attack categories (summarized in Table 1);

A communication overhead comparison, analyzing message size and transmission cost;

A computational overhead analysis, evaluating feasibility under resource-constrained EV and charging pile environments.

Through these additions, the revised evaluation demonstrates that PufCB-Auth provides stronger security coverage than either baseline scheme, particularly against long-term impersonation and behavior mimicry attacks, while maintaining comparable lightweight performance characteristics. Importantly, the comparison highlights that the security advantage of PufCB-Auth arises not merely from combining multiple factors, but from the careful integration of independent hardware- and behavior-derived identities into a unified digital fingerprint with continuous authentication capability.

**Revisions made:**

- Added a new subsection **“Threat Model and Security Evaluation Methodology”** to define unified adversarial assumptions.
- Added a **comparative security analysis** against PUF-MAP and L2AI under the same threat model.
- Introduced **Table 1** to summarize attack resistance across schemes.
- Added communication and computational overhead comparisons to demonstrate deployment feasibility.

These revisions can be found in **Sections 5.4–5.5 and Tables 1–2** of the revised manuscript.

**Major Comment 2**

The manuscript lacks a systematic threat model and formal security analysis.

**1.2 Response:**

We sincerely thank the reviewer for pointing out this important omission. We fully agree that a clear and systematic threat model, together with a structured security analysis, is essential for evaluating the robustness and credibility of any newly proposed authentication scheme.

In the original version of the manuscript, security-related discussions were limited to brief mentions of replay and modeling attacks, which was insufficient to provide a comprehensive understanding of the adversarial assumptions and the protection guarantees of the proposed framework. In response to this comment, we have significantly strengthened the manuscript by introducing a dedicated threat model and security evaluation methodology, followed by a systematic security analysis grounded in this model.

Specifically, we added a new subsection entitled “Threat Model and Security Evaluation Methodology”, in which we explicitly define the capabilities of a probabilistic polynomial-time adversary operating in an EV charging environment. The threat model formalizes the adversary’s abilities along five dimensions, including:

(1) full control over the public communication channel (eavesdropping, replay, and message manipulation),

(2) impersonation attempts targeting either EVs or charging piles,

(3) partial device compromise and leakage of stored parameters,

(4) long-term observation and manipulation of charging behavior data, and

(5) abuse of authentication failures or update mechanisms to induce denial-of-service or poisoning effects.

Based on this unified threat model, we then conducted a structured qualitative security analysis that evaluates the resistance of the proposed PufCB-Auth scheme against a comprehensive set of representative attacks, including replay attacks, man-in-the-middle attacks, impersonation attacks, partial device capture, behavior mimicry, long-term masquerading, and privacy leakage. Rather than claiming absolute security, the analysis focuses on identifying the adversarial conditions required for a successful compromise and highlights how multiple independent factors must be simultaneously defeated to break authentication.

Furthermore, to provide context and comparability, we applied the same threat model to two representative baseline schemes (PUF-MAP and L2AI). This allows a fair comparison of security coverage under identical adversarial assumptions and clearly demonstrates how PufCB-Auth improves long-term security robustness by combining hardware-rooted identity with continuous behavior-based verification.

This threat-model-driven analysis complements the experimental evaluation and clarifies the security rationale of the proposed design, particularly its ability to mitigate long-term impersonation and behavior mimicry attacks that are not adequately addressed by conventional one-time authentication schemes..

**Revisions made:**

- Added a new subsection **“Threat Model and Security Evaluation Methodology”** defining adversarial capabilities and security assumptions.
- Introduced a **systematic security analysis** covering replay, MITM, impersonation, partial device capture, behavior mimicry, privacy leakage, and long-term masquerading attacks.
- Added **Table 1** to summarize and compare attack resistance across PufCB-Auth and representative baseline schemes.

These revisions are included in **Section 5.4 and Section 5.5** of the revised manuscript.

**Major Comment 3**

The update mechanism in Enhanced PufCB-Auth is vulnerable to poisoning or DoS attacks, as malicious behavior could trigger profile updates.

**1.3 Response:**

We sincerely thank the reviewer for identifying this critical security issue. We fully agree that, if improperly designed, an anomaly-triggered update mechanism may introduce serious vulnerabilities, including update poisoning and denial-of-service (DoS) attacks. This comment is highly valuable and has led us to fundamentally redesign the fingerprint update mechanism in the revised manuscript.

In the original version, anomaly detection and feature update were conceptually coupled, which could indeed allow adversarial behaviors to indirectly influence the update process. In response, we have decoupled risk assessment from profile updating and introduced a set of strict admission and control policies to ensure that malicious or abnormal behaviors can never be incorporated into legitimate authentication baselines.

Specifically, the revised update mechanism adopts the following safeguards:

First, high-risk or anomalous charging behaviors are explicitly excluded from the update dataset. Such observations are used solely for risk assessment and security response, and are never admitted into the feature update process. Only historical charging records that have successfully passed authentication are eligible for updating the behavioral baseline.

Second, profile updates are subject to rate-limiting and controlled execution policies. Even when update conditions are met, updates are not performed immediately or repeatedly. If update triggers occur at an abnormally high frequency, the system escalates to an auxiliary verification stage instead of performing automatic updates, effectively preventing adversaries from inducing DoS attacks through repeated anomaly triggering.

Third, to accommodate legitimate long-term behavior evolution without sacrificing security, update operations are performed conservatively and only at controlled frequencies. This ensures that gradual, benign behavior drift can be captured, while sudden abnormal deviations—whether accidental or malicious—do not contaminate the authentication profile.

Fourth, for hardware-derived identity factors (PUF responses), updates are performed even more conservatively. Only recent authentication-passed records are used to compute updated physical characteristics. If significant deviations persist beyond acceptable tolerance even after incorporating legitimate data, the system interprets this as potential device replacement or impersonation rather than benign drift, and authentication is rejected instead of adaptively accommodated.

Through these design changes, the update mechanism no longer allows malicious behavior to be registered as legitimate identity features. Anomaly detection now strictly serves as a trigger for security escalation rather than an automatic learning signal, thereby eliminating the poisoning pathway identified by the reviewer.

**Revisions made:**

- Fundamentally redesigned the **digital fingerprint update mechanism** to decouple anomaly detection from profile updating.
- Introduced **strict admission control**, allowing only authentication-passed records to participate in updates.
- Added **rate-limiting and auxiliary verification mechanisms** to prevent update abuse and DoS attacks.
- Clarified conservative update policies for both **behavior-derived** and **PUF-derived** identity factors.

These changes are described in detail in **Section 3.4 (Enhanced PufCB-Auth Based on Continuous Digital Fingerprint Update)** of the revised manuscript.

**Major Comment 4**

The CB-Auth module relies on a 3σ rule for its dynamic threshold, which assumes a normal distribution. This assumption is questionable for charging behavior data, which is likely to be multi-modal or skewed. The authors should validate this assumption or adopt a more robust non-parametric method.

**1.4 Response:**

We sincerely thank the reviewer for this thoughtful and technically important comment. We agree that **raw EV charging behavior features**—such as charging time, location, and demand—are unlikely to follow a normal distribution and may indeed exhibit multi-modal or skewed characteristics. This observation is correct and well taken.

However, we would like to clarify that the proposed CB-Auth module **does not assume that raw charging behavior features follow a normal distribution**. In the revised manuscript, we have clarified both the design rationale and the statistical interpretation of the dynamic thresholding mechanism.

Specifically, the 3σ rule is **not applied to raw behavioral features**, but rather to the **user-specific deviation score** (Δ), which is computed as a weighted aggregation of negative log-probability values derived from each individual user’s historical behavior distribution. Each user’s deviation score sequence reflects the dispersion of how current behaviors deviate from that user’s own established behavioral profile, rather than from a global population model.

In this context, the mean (μΔ) and standard deviation (σΔ) are calculated over a sliding window of recent deviation scores for each individual user. The resulting threshold τΔ = μΔ + 3σΔ therefore serves as an **adaptive, user-specific risk boundary**, rather than a statistical assumption about the distribution of charging behavior itself. When a user’s behavior is stable, the deviation score distribution is tightly concentrated, and all observations may fall well below the threshold. When behavior becomes abnormal, deviation scores increase sharply and cross the adaptive boundary.

We have further clarified in the revised manuscript that the proposed method **does not force a fixed proportion of observations to be labeled as anomalous**, which is a known limitation of some non-parametric methods such as percentile- or IQR-based thresholds. Instead, the variance-adaptive thresholding strategy allows all observations to be treated as low risk when user behavior is stable, which better aligns with continuous authentication requirements in real-world charging scenarios.

To address the reviewer’s concern more explicitly, we have revised the manuscript to clearly state that:

- The normality assumption does **not** apply to raw charging behavior features;
- The threshold operates on **user-specific deviation scores**, not on absolute behavior values;
- The method is designed to adapt to different levels of behavioral variability across users.

**Revisions made:**

- Clarified the statistical interpretation of the **3σ-based dynamic threshold**, emphasizing that it applies to deviation scores rather than raw behavior features.
- Explicitly stated that **no normal distribution assumption** is made for EV charging behavior data.
- Added discussion explaining why variance-adaptive thresholding is preferred over fixed percentile-based methods in continuous authentication contexts.

These clarifications are included in **Section 3.2 (CB-Auth: A User-level EV-pile Authentication Based on EV Charging Behavior Features)** of the revised manuscript.

**Minor Comments:**

**Minor Comment 1**

**Reviewer’s comment:**

*The experiments rely on simulated data. The authors should provide the simulation scripts and parameters to ensure reproducibility.*

**Response:**

We thank the reviewer for this important comment regarding reproducibility. We agree that transparency of the simulation setup and parameter configuration is essential, especially when real-world data cannot be fully disclosed due to practical or confidentiality constraints.

In the revised manuscript, we have substantially expanded the description of the experimental platform, simulation procedures, and parameter settings. The simulation environment now clearly specifies how PUF-like responses are generated, how noise and drift are modeled, and how charging behavior data are synthetically constructed to emulate long-term user patterns under different conditions. These details enable independent researchers to reproduce the experimental results and validate the conclusions.

In addition, we clarify that the experimental platform is designed to evaluate the **authentication framework and its robustness to noise and behavior drift**, rather than to benchmark a specific commercial PUF circuit or proprietary dataset.

**Revisions made:**

- Added detailed descriptions of simulation assumptions, data generation procedures, and parameter settings in **Appendix B** of the revised manuscript.

**Minor Comment 2**

**Reviewer’s comment:**

*The manuscript uses several “magic numbers” without sufficient justification (e.g., PUF similarity threshold τ = 0.95, behavior window size S = 100, and risk threshold 0.3τΔ).*

**Response:**

We thank the reviewer for highlighting this issue. We acknowledge that these parameters were not sufficiently explained in the original manuscript.

In the revised version, we clarify that these parameters are **empirically selected** to demonstrate the feasibility and stability of the proposed framework under typical operating conditions. The selected values are consistent with commonly used thresholds in related PUF-based authentication and behavior-based anomaly detection literature, and were chosen to balance robustness and sensitivity in a resource-constrained EV charging environment.

We further emphasize that these parameters are **not fixed system constants** and can be tuned according to deployment requirements, environmental noise levels, and user behavior variability. A systematic sensitivity analysis and adaptive parameter optimization are identified as important directions for future work.

**Revisions made:**

- Added explanatory text discussing the rationale and flexibility of key parameter choices in the **Experimental Evaluation** section.

**Minor Comment 3**

**Reviewer’s comment:**

*Figures 3 and 4 are of poor resolution, with inconsistent and difficult-to-read axis labels. There is also a copy-paste error in the figure captions.*

**Response:**

We thank the reviewer for pointing out these presentation issues. We agree that figure quality and clarity are important for effective communication of experimental results.

All figures have been carefully reviewed and revised. Figures 3 have been redrawn with higher resolution, consistent formatting, and clearer axis labels. In addition, the caption numbering and textual descriptions have been thoroughly checked and corrected to eliminate copy-paste errors.

**Revisions made:**

- Redrew and reformatted all affected figures with improved resolution and consistent labeling.
- Corrected figure captions and numbering throughout the manuscript.

**Minor Comment 4**

**Reviewer’s comment:**

*User-dependent variability is observed in the experimental results (e.g., differences between User C and User E).*

**Response:**

We thank the reviewer for this observation. In the revised manuscript, we clarify that such user-dependent variability reflects **natural differences in charging behavior stability and regularity across users**, rather than a flaw in the authentication framework. Users with more regular charging habits naturally yield tighter behavior profiles and higher authentication stability, while users with more irregular patterns exhibit greater variance.

Importantly, the proposed framework is designed to adapt to such variability through user-specific baselines and dynamic thresholds, rather than enforcing a uniform global model. This design choice enhances fairness and robustness in real-world deployments involving heterogeneous user populations.

**Revisions made:**

- Added clarifying discussion on user-dependent variability in the **Results and Discussion** section.

**Response to Reviewer 2’s Comments**

**Comments:**

**2.0 Response:**

We sincerely thank Reviewer #2 for the positive evaluation of our work and for the constructive and detailed comments. The reviewer’s suggestions have helped us clarify design choices and improve the presentation of the proposed framework. Our point-by-point responses are provided below.

**Comment 1:**

The paper proposes a lightweight multi-factor continuous authentication scheme for EV charging scenarios. Please further clarify the motivation and core contributions, especially in the context of high-power ChaoJi charging.

**2.1 Response:**

We thank the reviewer for this helpful suggestion. In response, we have strengthened the motivation and clarified the core contributions of the work in the Introduction.

In the revised manuscript, we explicitly emphasize that ultra-high-power charging scenarios enabled by the ChaoJi standard introduce stricter security and safety requirements. Authentication failures in such contexts may not only lead to unauthorized access but can also cause equipment overload, thermal stress, or even grid-level disturbances. These risks motivate the need for authentication mechanisms that are not only secure but also lightweight, adaptive, and capable of continuous verification during the charging process.

We further clarify that the key contribution of this work lies not simply in combining multiple authentication factors, but in **integrating hardware-rooted identity and user behavior into a unified digital fingerprint with continuous authentication and adaptive update capability**, tailored for resource-constrained EV–pile interaction environments.

**Revisions made:**

- Revised and expanded the Introduction to better articulate the motivation and contributions in ChaoJi charging scenarios.

**Comment 2:**

How are the weights assigned to different charging behavior features (e.g., time, location, demand)?

**2.2 Response:**

We thank the reviewer for this question. In the current work, the weights assigned to different behavior features are determined through **offline statistical analysis of historical charging data**, considering each feature’s contribution to identity discrimination and stability. The weighting strategy is designed to balance sensitivity and robustness across heterogeneous behavior dimensions.

We note that the primary objective of this paper is to validate the feasibility and effectiveness of the proposed **multi-factor authentication framework**, rather than to introduce a new adaptive weighting algorithm. To avoid over-claiming, we have clarified this design choice in the revised manuscript and explicitly identify **adaptive or user-specific weight learning** as an important direction for future work.

**Revisions made:**

- Added clarification on feature weight determination and future extensions in **Section 3.2**.

**Comment 3:**

How is the size of the challenge pool determined, and is there a risk of exhausting it over the vehicle's lifetime?

**2.3 Response:**

We thank the reviewer for raising this point. In the proposed scheme, the challenge space is **not statically enumerated**.

Instead, challenges are dynamically constructed using perturbation-based generation and hash-chain expansion mechanisms. As a result, the effective challenge space is orders of magnitude larger than the number of authentication sessions expected over a vehicle’s operational lifetime.

Therefore, challenge pool exhaustion is not a practical concern under realistic deployment conditions. This clarification has been added to the revised manuscript.

**Comment 4:**

The update mechanism is triggered by a high anomaly score. Could this be exploited by an attacker to force frequent, unnecessary updates, potentially leading to a Denial-of-Service (DoS) by overwhelming the system?

**2.4 Response:**

We thank the reviewer for this valuable observation. We agree that update mechanisms in behavior-aware authentication systems must be carefully designed to avoid security risks such as poisoning or denial-of-service.

As clarified and revised in the manuscript, the proposed update mechanism **does not directly learn from anomalous or high-risk behaviors**. Instead, anomaly detection is used purely as a trigger for security escalation, while **only authentication-passed historical records** are eligible for profile updates. In addition, update operations are rate-limited and subject to controlled execution policies.

These safeguards ensure that malicious or abnormal charging behaviors cannot be incorporated into legitimate authentication baselines, while still allowing the system to adapt to long-term benign behavior drift.

**Revisions made:**

- Clarified the separation between anomaly detection and profile updating in **Section 3.4**.

**Comment 5:**

How does the scheme handle the initial "cold start" problem for new EV users who have little to no historical charging data to establish a reliable behavioral baseline?

**2.5 Response:**

We thank the reviewer for this practical question.

In the cold-start phase, new users initialize their profiles through a registration process in which PUF features are enrolled and an initial behavioral baseline is established. This baseline can be constructed either from the user’s own early charging records or, when appropriate, from aggregated profiles of similar user types.

As more authenticated charging sessions are accumulated, the behavior profile is gradually refined through the controlled update mechanism, allowing the system to adapt without compromising security.

**Comment 6:**

The communication overhead is calculated for authentication, but what is the total overhead during the initial registration phase of a new EV with the charging infrastructure?

**2.6 Response:**

We thank the reviewer for this question. Registration is a **one-time or low-frequency operation** performed prior to normal charging interactions. It does not affect real-time charging performance or safety, especially in ultra-fast charging scenarios where authentication latency is critical.

Accordingly, our performance evaluation focuses on the authentication phase rather than the registration phase. This design choice has been clarified in the revised manuscript.

**Comment 7:**

How is the integrity and authenticity of this update process ensured to prevent a man-in-the-middle attacker from injecting a malicious new fingerprint?

**2.7 Response:**

We thank the reviewer for this important question. Fingerprint updates are protected using authenticated and encrypted communication channels. Only verified update operations are permitted to replace existing fingerprints, and all update events are securely logged to ensure traceability and prevent unauthorized modification.

This mechanism prevents man-in-the-middle attacks or malicious profile injection during the update process.

**Comment 8:**

What type of PUF is simulated in the experimental evaluation?

**2.8 Response:**

We thank the reviewer for requesting this clarification. The experimental platform emulates a **PUF-like hardware fingerprint derived from BMS analog characteristics**, rather than implementing a specific silicon PUF primitive (e.g., SRAM PUF or RO PUF). This choice allows us to evaluate the robustness of the proposed authentication framework under realistic noise and drift conditions, without being tied to a particular hardware implementation.

We have explicitly stated this design choice in the revised manuscript to avoid ambiguity.

**Comment 8:**

Explicitly stating the assumed adversary's capabilities (e.g., can they physically tamper with the BMS? Can they observe network traffic?) would better frame the security claims and highlight which specific threats PufCB-Auth is designed to mitigate.

**2.9 Response:**

We thank the reviewer for this helpful suggestion. In response, we have added a **dedicated threat model and security evaluation methodology** to the revised manuscript. The threat model explicitly defines adversarial capabilities relevant to EV charging environments, including network-level attacks, impersonation attempts, partial device compromise, long-term behavior observation, and update abuse.

This unified threat model serves as the foundation for the subsequent security analysis and comparative evaluation, ensuring that all schemes are assessed under consistent and realistic assumptions.

**Revisions made:**

- Added a new **Threat Model and Security Evaluation Methodology** subsection in **Section 5.4**.

**Response to Reviewer 3’s Comments**

**Comments:**

**3.0 Response:**

We sincerely thank Reviewer #3 for the constructive comments and helpful suggestions. The reviewer’s feedback has assisted us in improving the clarity, positioning, and presentation of the manuscript. Our detailed responses are provided below.

**Comment 1:**

The title of the manuscript is overly long and could be shortened to improve readability.

**3.1 Response:**

We thank the reviewer for this helpful suggestion. We agree that the original title was overly long and could reduce readability.

In the revised manuscript, the title has been shortened and refined to better balance clarity and technical accuracy, while still conveying the core contributions of the work.

**Revisions made:**

- Revised and shortened the manuscript title.

**Comment 2:**

The manuscript would benefit from clearer positioning relative to existing work and more structured comparison with related approaches.

**3.2 Response:**

We thank the reviewer for this suggestion. In response, we have strengthened the related work discussion and improved the positioning of the proposed scheme relative to existing authentication approaches.

The revised manuscript now includes structured comparisons in the security analysis and performance evaluation sections, which more clearly differentiate the proposed approach from representative state-of-the-art schemes.

**Revisions made:**

- Added structured comparisons in the Security Analysis and Experimental Evaluation sections.

**Comment 3:**

lacks a security assessment of the proposed multi-factor authentication scheme*.*

**3.3 Response:**

We agree with the reviewer that a comprehensive security assessment is essential. In response, we introduced a dedicated Threat Model and Security Evaluation Methodology section and conducted a systematic security analysis under explicitly defined adversarial assumptions.

This analysis evaluates the proposed scheme against representative attack categories, including replay attacks, man-in-the-middle attacks, impersonation, partial device capture, behavior mimicry, and long-term masquerading. The results demonstrate that the proposed framework provides broader security coverage than conventional one-time authentication schemes.

**Revisions made:**

- Added a new Threat Model and Security Evaluation Methodology subsection.
- Expanded the Security Analysis section.

Comment 4:

Please provide a comparative evaluation between the proposed solution and existing related solutions in terms of security and execution performance, so that the conclusion can be more convincing..

**3.4 Response:**

We thank the reviewer for this comment. In the revised manuscript, we have added a comparative evaluation against representative lightweight authentication schemes, including both hardware-centric and behavior-centric approaches. The comparison covers security capability, communication overhead, and computational cost, providing a more complete assessment of the proposed method’s advantages and trade-offs.

**Revisions made:**

- Added comparative evaluation tables and discussion in Sections 5.4–5.5.
